# Supplementary material for: Longitudinal cardio-respiratory fitness prediction through wearables in free-living environments
Source: NPJ Digit Med. 2022 Dec 2;5:176. doi: 10.1038/s41746-022-00719-1 (PMC9718831; doi:10.1038/s41746-022-00719-1)
Supplement: Supplementary file 1 — Supplementary Information [file 41746_2022_719_MOESM1_ESM.pdf]

Supplementary information

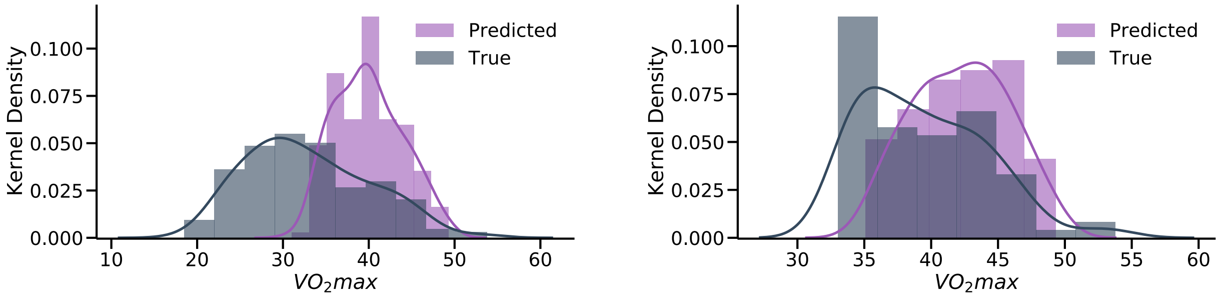

(a) **(b)**

Figure Suppl. 1. External validation of Fenland I model with maximal (peak exercise) test data using the BBVS cohort. **(a)** Distribution of the predicted vs the true VO2max (RMSE=8.998) using all participants (N=181). **(b)** Distribution of the predicted vs the true VO2max (RMSE=5.19) by matching BBVS to have similar VO2max (mean±std) to the training set of Fenland I, using a subset of participants (N=82). Please see the main text for interpretation of these results.

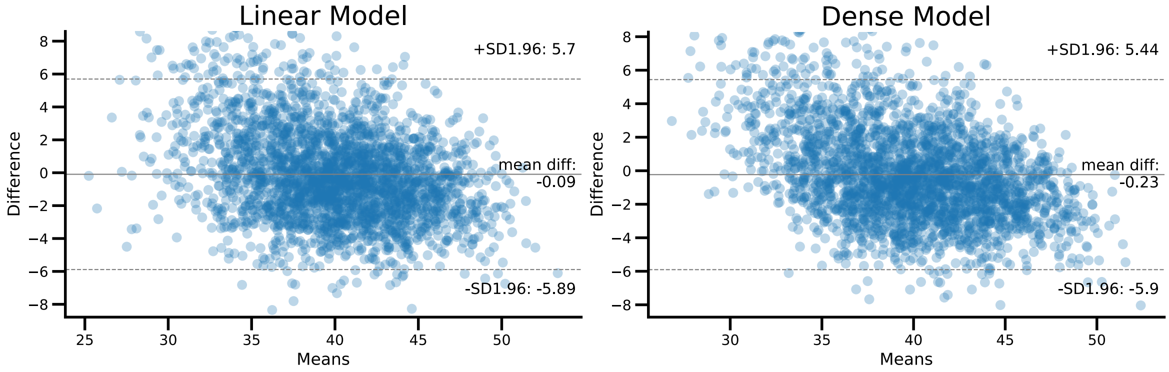

(a) Linear regression **(b)** Feature-based Dense neural network

Figure Suppl. 2. Bland–Altman plots of the comprehensive linear and Dense models. (a-b) Both models demonstrate low bias and high agreement between true and predicted values, with the Dense model showing lower standard deviation on the top values.

Features/Variables	Description
**Sensors**	
Acceleration*	Acceleration measured in mg
Heart rate (HR)*	Mean HR resampled in 15sec intervals, measured in BPM
Heart Rate Variability (HRV)*	HRV calculated by differencing the second-shortest and the second-longest inter-beat interval (as seen in[34]), measured in ms
Acceleration-derived Euclidean Norm Minus One (ENMO)*	ENMO-like variable (Acceleration/0.0060321) + 0.057) (as seen in[35])
Acceleration-derived Metabolic Equivalents of Task (METs)*	
Sedentary*	If Accelerometer <1, take daily count and average
Moderate to Vigorous*	If Accelerometer >= 1, take daily count and average
Vigorous*	If Accelerometer >= 4.15, take daily count and average
**Anthropometrics**	
Age	Age, measured in years
Sex	Sex is binary (female/male)
Weight	Weight, measured in kilograms
Height	Height, measured in meters.centimeters
Body Mass Index (BMI)	BMI is calculated by Weight/($Height$ˆ2), measured in kg$/m$ˆ2
**Resting Heart Rate**	
Wearable-derived RHR	RHR is calculated by averaging the 4th, 5th, and 6th minute of the baseline visit and adding to that the Sleeping Heart Rate that has been inferred by the wearable device.[4]
**Seasonality**	
Month of year	The month number is used along with a coordinate encoding that allows the models to make sense of their cyclical sequence.

Table Suppl. 2. Description of the features/variables used in our analysis as inputs to the models. The features with asterisks(*) are time-series and therefore we have extracted the following statistical variables: *mean, minimum, maximum, standard deviation, percentiles (25%, 50%, 75%), and the slope of a linear regression fit*. The final set of features is 68.
