# Supplementary material for: Longitudinal cardio-respiratory fitness prediction through wearables in free-living environments
Source: NPJ Digit Med. 2022 Dec 2;5:176. doi: 10.1038/s41746-022-00719-1 (PMC9718831; doi:10.1038/s41746-022-00719-1)
Supplement: Supplementary file 2 — Reporting Summary [file 41746_2022_719_MOESM2_ESM.pdf]

nature portfolio

Corresponding author(s): Dimitris Spathis

Last updated by author(s): Sep 19, 2022

Reporting Summary

Nature Portfolio wishes to improve the reproducibility of the work that we publish. This form provides structure for consistency and transparency in reporting. For further information on Nature Portfolio policies, see our Editorial Policies and the Editorial Policy Checklist.

Statistics

For all statistical analyses, confirm that the following items are present in the figure legend, table legend, main text, or Methods section.

n/a	Confirmed	
☐	☒	The exact sample size (*n*) for each experimental group/condition, given as a discrete number and unit of measurement
☒	☐	A statement on whether measurements were taken from distinct samples or whether the same sample was measured repeatedly
☐	☒	The statistical test(s) used AND whether they are one- or two-sided<br>*Only common tests should be described solely by name; describe more complex techniques in the Methods section.*
☐	☒	A description of all covariates tested
☒	☐	A description of any assumptions or corrections, such as tests of normality and adjustment for multiple comparisons
☐	☒	A full description of the statistical parameters including central tendency (e.g. means) or other basic estimates (e.g. regression coefficient) AND variation (e.g. standard deviation) or associated estimates of uncertainty (e.g. confidence intervals)
☐	☒	For null hypothesis testing, the test statistic (e.g. *F*, *t*, *r*) with confidence intervals, effect sizes, degrees of freedom and *P* value noted<br>*Give P values as exact values whenever suitable.*
☒	☐	For Bayesian analysis, information on the choice of priors and Markov chain Monte Carlo settings
☒	☐	For hierarchical and complex designs, identification of the appropriate level for tests and full reporting of outcomes
☒	☐	Estimates of effect sizes (e.g. Cohen's *d*, Pearson's *r*), indicating how they were calculated

Our web collection on statistics for biologists contains articles on many of the points above.

Software and code

Policy information about availability of computer code

Data collection	All preparation and analysis of data from the Fenland cohort were done at the MRC Epidemiology Unit and the Department of Computer Science and technology, both at the University of Cambridge, UK.
Data analysis	All analyses were undertaken in Python v. 3.6.0. Predictive models and figures were produced with the following software libraries: matplotlib v. 3.2.2, pandas v. 1.0.5, scikit-learn v. 0.23.1, numpy v. 1.19.0, tensorflow v. 1.4.0, and keras v. 2.2.0, as stated in the manuscript. The researchers wrote their own code for these analyses. Details and references can be found in the relevant Methods sections.

For manuscripts utilizing custom algorithms or software that are central to the research but not yet described in published literature, software must be made available to editors and reviewers. We strongly encourage code deposition in a community repository (e.g. GitHub). See the Nature Portfolio guidelines for submitting code & software for further information.

Data

Policy information about availability of data

All manuscripts must include a data availability statement. This statement should provide the following information, where applicable:

- Accession codes, unique identifiers, or web links for publicly available datasets
- A description of any restrictions on data availability
- For clinical datasets or third party data, please ensure that the statement adheres to our policy

All Fenland data used in our analyses is available from the MRC Epidemiology Unit at the University of Cambridge upon reasonable request (http://www.mrc-epid.cam.ac.uk/research/studies/fenland/).

Field-specific reporting

Please select the one below that is the best fit for your research. If you are not sure, read the appropriate sections before making your selection.

☒ Life sciences ☐ Behavioural & social sciences ☐ Ecological, evolutionary & environmental sciences

For a reference copy of the document with all sections, see nature.com/documents/nr-reporting-summary-flat.pdf

Life sciences study design

All studies must disclose on these points even when the disclosure is negative.

Sample size	We use individual-level data from 11,059 participants of the Fenland cohort with available sensor, demographic, and cardiorespiratory fitness data.
Data exclusions	From the initial 12,318 original participants of the Fenland I study we excluded 160 due to insufficient number of days of sensor data and another 1,099 for not completing the baseline visit. We describe all data exclusion steps in FIgure 6.
Replication	We replicated our results in multiple held-out testing sets. As it is common in machine learning experiments, we evaluate the performance of trained predictive models on unseen testing sets. For the main task of the paper (Task 1), the testing set consists of 2,675 participants.
Randomization	The participants were allocated to training and testing sets based on whether they provided longitudinal data. We split participant data into independent training and test sets. The training set (n=8384, participants with baseline data only) was used for model development. The testing set (n=2675, participants with baseline and followup data) was used to externally validate each model. This allows to re-use the trained model from Task 1, with different sensor data from Fenland II participants. Intuitively, we train a model on the big population, and we evaluate it with two snapshots of another longitudinal population over time (Tasks 1 & 3). The covariates were not explicitly controlled due to the high-dimensional covariate space (68 covariates). We list some key demographics of all groups in Table 1, along with the mean and std.
Blinding	Blinding does not apply as information of group allocation was needed to perform the analyses.

Reporting for specific materials, systems and methods

We require information from authors about some types of materials, experimental systems and methods used in many studies. Here, indicate whether each material, system or method listed is relevant to your study. If you are not sure if a list item applies to your research, read the appropriate section before selecting a response.

Materials & experimental systems

n/a	Involved in the study	
☒	☐	Antibodies
☒	☐	Eukaryotic cell lines
☒	☐	Palaeontology and archaeology
☒	☐	Animals and other organisms
☐	☒	Human research participants
☒	☐	Clinical data
☒	☐	Dual use research of concern

Methods

n/a	Involved in the study	
☒	☐	ChIP-seq
☒	☐	Flow cytometry
☒	☐	MRI-based neuroimaging

Human research participants

Policy information about studies involving human research participants

Population characteristics	Fenland participants were on average 47 years old (standard deviation 7 years) and 52% female. A detailed presentation of the population is available in Table 1.
Recruitment	Fenland is a population-based cohort of 12,435 participants without diabetes born between 1950 and 1975. A detailed description can be found in the Methods section.
Ethics oversight	All participants provided written informed consent and the study was approved by the University of Cambridge, NRES Committee - East of England Cambridge Central committee (Ref 04/Q0108/19). All experiments and data collected were done in accordance with the declaration of Helsinki.

Note that full information on the approval of the study protocol must also be provided in the manuscript.
